# Evaluation of Success of Arterial Cannulation Employing the Dorsalis Pedis Artery Versus Posterior Tibial Artery: A Clinical Comparative Study

**DOI:** 10.5152/TJAR.2023.22826

**Published:** 2023-02-01

**Authors:** Rudrashish Haldar, Tapas Kumar Singh, Priyam Saikia, Ashish Kumar Kannaujia, Prabhaker Mishra, Anil Agarwal

**Affiliations:** 1 Department of Anaesthesia, Sanjay Gandhi Post Graduate Institute of Medical Sciences, Lucknow, Uttar Pradesh, India; 2 Department of Anaesthesia, Gauhati Medical College, Guwahati, India

**Keywords:** Arterial cannulation, dorsalis pedis artery, invasive blood pressure, palpation, posterior tibial artery

## Abstract

**Objective:**

The dorsalis pedis artery and posterior tibial artery are recognised sites for arterial cannulation. This study aimed to compare the first-attempt success rates of cannulation along with other cannulation characteristics of these 2 arteries in adult patients undergoing surgery under general anaesthesia using the conventional palpatory method.

**Methods:**

Two hundred twenty adults were allocated randomly into 2 groups. The dorsalis pedis artery and posterior tibial artery were attempted for cannulation in the dorsalis pedis artery and posterior tibial artery group, respectively. First-attempt success rates, cannulation times, number of attempts, ease of cannulation, and complications were recorded.

**Results:**

Demographic characteristics, pulse characteristics, single-attempt success rates, ease of cannulation, reasons for failure, and complications were similar. Single-attempt success rates were similar (64.5% and 61.8%, *P* = .675) with equal median attempt. Easy cannulation (Visual Analogue Scale score ≤4) was the same in both groups, whereas percentages of difficult cannulation (Visual Analogue Scale scores ≥4) were 16.4% and 19.1% in the dorsalis pedis artery and posterior tibial artery groups, respectively. Cannulation time was lower in the dorsalis pedis artery group [median time in seconds: 37 (28, 63) seconds vs. 44 (29, 75) seconds, *P* = .027]. Single-attempt success rates were lower in the feeble pulse group as compared to the strong pulse group (48.61% vs. 70.27%, *P* = .002). Likewise, a higher Visual Analogue Scale of ease of cannulation (>4 score) was seen in the feeble pulse group compared to the strong pulse group (26.39% vs. 13.51%, *P* = .019).

**Conclusions:**

The single-attempt success rate was similar for both dorsalis pedis artery and posterior tibial artery. However, the time taken for cannulating the posterior tibial artery is significantly higher than that for dorsalis pedis artery.

**Main Points**

- Both dorsalis pedis artery (DPA) and the posterior tibial artery (PTA) are recognised sites for arterial cannulation for periprocedural invasive monitoring and blood sampling.
- Both arteries can be cannulated using the blind palpatory method.
- The success rate of arterial cannulation in a single attempt was similar in both the groups.
- The time of cannulation was significantly less in the DPA group as compared to the PTA group. The incidence of complications was similar in both groups.

## Introduction

Arterial cannulation is frequently performed in the operation theatre (OT) and intensive care unit settings which aids in the definitive monitoring of blood pressure and evaluating respirophasic variations to assess fluid responsiveness. Arterial cannulation also provides a source of continuous sampling for various biochemical and physiological parameters.

The common vessels for cannulation include radial, ulnar, brachial, axillary, femoral, dorsalis pedis, and posterior tibial arteries. Most of the available literature on arterial cannulation focuses on upper extremity vessels (radial and ulnar)^1^ which are preferentially chosen considering their proximity to skin surface, accessibility, ease of placement, presence of collateral flow, and low risk of complications.^2,3^ However, conditions like burn or injury on the upper limbs, proximity to the surgical field, following repeated failed attempts to cannulate arteries of upper limbs, surgeries where access to the upper limb is curtailed or it cannot be immobilised prevent the use of upper limbs for arterial cannulation. This mandates the use of alternative vessels in the lower limbs for cannulation. The feasibility of using dorsalis pedis artery (DPA) for cannulation compared to the radial artery has been studied before.^4,5^ Similarly, the posterior tibial artery (PTA), though infrequently employed, is also an acceptable site on the lower limb,^6,7^ but its cannulation characteristics in adults have not been explored. To the best of our knowledge, no study has compared arterial cannulation characteristics of DPA versus PTA when accomplished using a blind palpatory method. We hypothesised that the cannulation characteristics of both arteries are similar and undertook this study with the primary objective of comparing their first-attempt success rates. Secondary objectives included a number of attempts, time taken, subjective ease of cannulation, and incidence of complications.

## Methods

After obtaining Institutional Ethics Committee approval of the Sanjay Gandhi Post Graduate Institute of Medical Sciences, Lucknow, India (2020-175-IP-115 dated 6 July 2020), registering the trial with the Clinical Trial Registry of India (CTRI/2020/07/026762 [Registered on: 24/07/2020]), and obtaining written informed consent from eligible patients, 239 adult patients were included in this prospective randomised patient and data analysis blinded, parallel-group study using consecutive sampling. The study was conducted between 10 August 2020 and 1 February 2021 in the OTs of SGPGI, Lucknow, India. Enrolled patients were given the option to withdraw themselves from the study at any moment without stating any reason. Patients (18-65 years) of either gender, belonging to the American Society of Anesthesiologists (ASA) physical status I or II and requiring invasive blood pressure monitoring during their intraoperative periods were eligible for inclusion. Patients refusing to participate, having skin erosions near the insertion site, diabetes mellitus, insufficient compensatory blood flow, Raynaud syndrome, coagulopathy, vascular diseases, and obese patients (body mass index (BMI) >30 kg m^−2^) were excluded.

The sample size was calculated based on a previous study where the reported first-pass success rates of cannulation of DPA by the palpatory method were 60%.^8^ To detect a difference of 20%, with a confidence level of 95% and a power of 80%, 95 patients were needed in each group. To account for study errors or attrition, we included 110 patients in each group. Based on a computer-generated sequence of randomisation (obtained from http://www.randomization.com), a 1:1 group allocation was done and was kept concealed in opaque sealed envelopes which were opened just prior to arterial cannulation by an anaesthesia technician who was not involved in the study.

The patients were briefed about the study protocol, and willing patients were recruited after obtaining written and informed consent in English or Hindi by the investigators (R.H., T.K.S., and A.K.K.). Enrolled patients were treated with the highest ethical standards in accordance with the Declaration of Helsinki. Patients were screened for the presence of palpable pulsation at the DPA and PTA of both lower limbs during the pre-anaesthetic checkup. Patency of foot collateral circulation was screened as per the test suggested by Johnstone and Greenhow^9^ where the DPA is compressed with external pressure to blanch the great toenail and thereafter observing its flushing as the blood returns. Rapid return of colour suggests adequacy of the lateral plantar collateral circulation. Both feet were tested as circulation is often not symmetrical bilaterally and only patients with patent bilateral circulation were chosen. Despite its limitations during vasoconstriction or when feet are cold, this test has been found to be clinically appropriate.^4^ The anaesthesia protocol was standardised for all patients. Anaesthesia induction was achieved with propofol (1.5-2.5 mg kg^−1^) and fentanyl (2 µg kg^−1^). Tracheal intubation was facilitated with vecuronium (0.1 mg kg^−1^). For intraoperative maintenance, a mixture of air and oxygen (FiO_2_ 0.5%) along with sevoflurane (1%-2%) and intermittent boluses of vecuronium were used. Depending upon allocation, either DPA or PTA was attempted to be cannulated. A single anaesthesiologist with prior experience of more than 100 DPA and PTA cannulations performed all the procedures to negate interindividual variability. The palpation-guided simple catheter over needle technique using a 20-G arterial cannula (BD 20G/1.10 mm × 45 mm, 49 mL min^−1^, Becton Dickinson Infusion Therapy System Inc. Utah, USA) with flow switch was performed. Cannulation was attempted 10 minutes after endotracheal intubation when haemodynamic parameters had stabilised and when no vasopressors were being used. The area was cleaned with chlorhexidine 2% and draped to maintain strict asepsis. Then, the designated artery of the nondominant lower limb was palpated with a gloved hand and the point of maximum pulsation was located. The pulsation was then graded as strong, feeble, or absent. The site of maximum pulsation was usually the most dorsal prominence of the navicular bone for DPA.^10^ The foot was dorsiflexed at 90° and externally rotated at a point which is one-third between the point of the medial malleolus and the point of the heel during PTA cannulation.^6,11^ Patients with either strong or feeble pulsation were then chosen for arterial cannulation. An alternative site was chosen in patients with absent pulsation, and they were excluded from the analysis. After palpating the point of maximum pulsation of the respective artery, the cannula was inserted at an angle of 30° to 45° to the skin and then advanced over the needle till arterial blood flashback was observed through the cannula’s hub. The catheter was then threaded inside the arterial lumen. The inserted catheter was now connected to a closed blood withdrawal device and a pressure transducer set and fixed on the skin with a sterile adhesive dressing. Time to successful arterial cannulation (T1) was defined as the time from starting palpation to the proper placement of the 20G cannula, confirmed by the appearance of an arterial waveform on the monitor. The number of cannulation attempts was quantified as “the number of times the needle has to be advanced through a new skin puncture.” The success rate was defined as “successful cannulation of the artery in 3 attempts or less.” The number of cannulation attempts was limited to 3 attempts and patients who experienced 3 failed attempts were labelled as failures. If before 3 attempts, haematoma developed or the pulsation was obliterated, further attempts at that particular site were abandoned and that procedure too was termed as “failure.” In cases of failure, alternative sites for arterial cannulation were chosen and the patients were excluded. All these parameters were recorded by an anaesthesia technician not involved in the study. The proceduralist rated the ease of cannulation on a Visual Analogue Scale (VAS) of 1-10 (1 easiest and 10 being the most difficult). For the purpose of analysis, VAS scores ≤4 were graded as “easy cannulation,” whereas scores >4 were graded as “difficult cannulation.”

After the conclusion of surgery, prior to shifting the patient, the arterial cannula was removed aseptically, and direct pressure was applied at the exit point of the catheter for 5 minutes. If the clinical condition dictated, the cannula was retained for monitoring purposes in the postoperative period. In either case, the total time the indwelling cannula remained intraarterially was noted. The patients were followed up for 5 days postoperatively and any complications like digital ischaemia, haemorrhage, haematoma formation, infection, abnormal skin colour, or neurologic abnormalities were recorded and suitably treated. Though the proceduralist was not blinded, the code was maintained till the completion of data analysis.

Continuous variables are represented as mean ± standard deviation/median (Q1, Q3), whereas categorical variables in frequency (%). Comparison between the 2 groups was done using independent samples *t*-test for means, Mann–Whitney *U*-test for median, and proportions by chi-square test-. Comparison between the 2 groups is presented by box plot (minimum, Q1, median, Q3, maximum) and adjacent bar diagram (frequency, %). *P* < .05 was considered statistically significant. Statistical analyses were performed using Statistical Package for Social Sciences, version 23.0 (IBM Corp.; Armonk, NY, USA).

## Results

**Figure 1.**
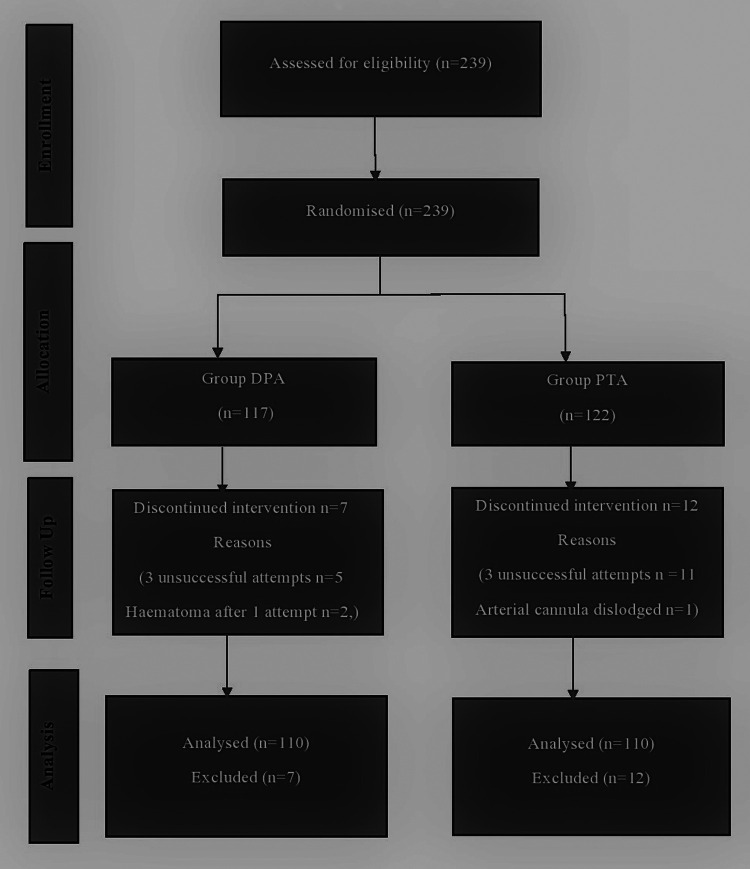
CONSORT flow chart.

**Figure 2.**
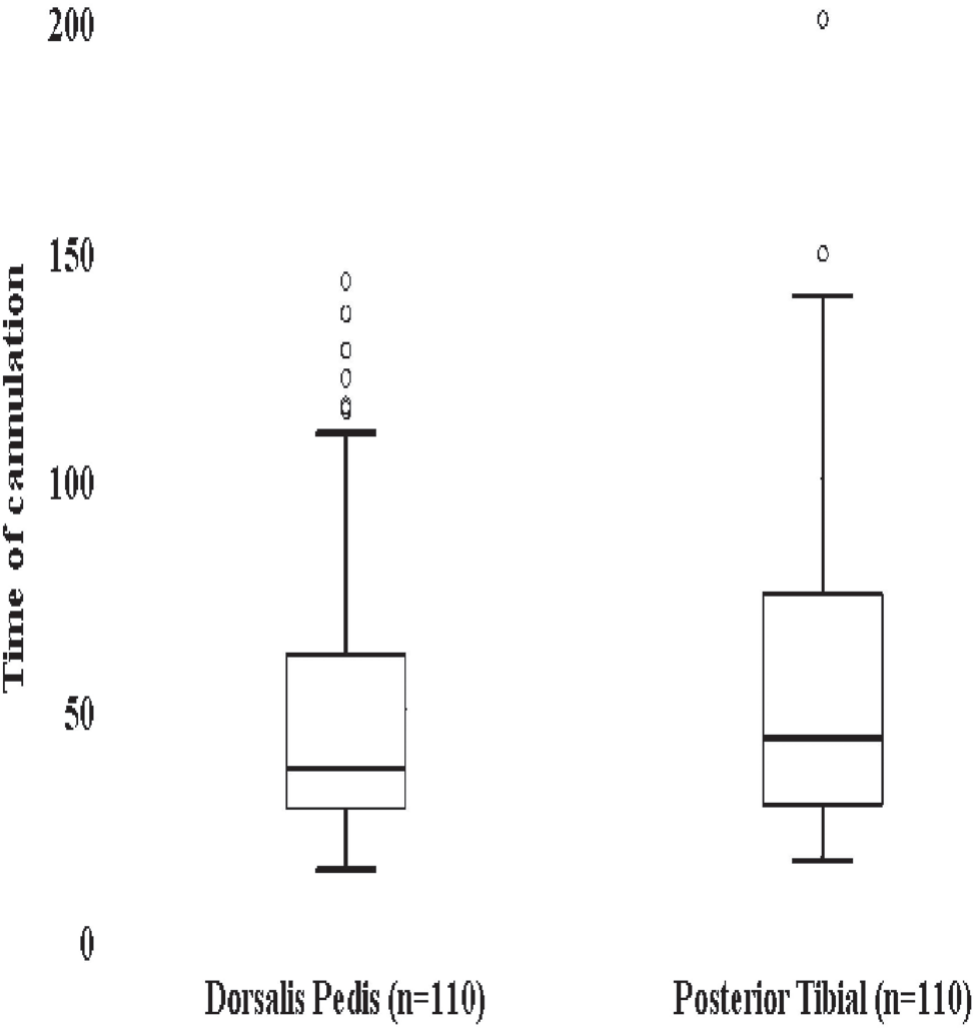
Box plot showing the distribution of time of cannulations (seconds) of the study patients in terms of their minimum, 25th, 50th, 75th percentile, and maximum value (lower to upper direction, respectively), whereas dot points outside the box plot indicate the extreme values of the data.

A total of 239 patients were included in this study, out of which 19 patients were excluded. In 110 patients, DPA was cannulated, whereas in another 110, patients’ PTA was cannulated. The CONSORT flow chart is shown in Figure 1. Baseline characteristics, number of attempts, cannulation time, ease, and complications were compared between the 2 groups. Age and BMI were comparable, whereas there were more male participants in the PTA group (Table 1). Most patients belonged to ASA grade I (67.3% and 59.1%) in DPA and PTA groups, respectively (Table 1). The majority of patients in both groups had strong pulses (70.9% in DPA and 63.6% in PTA groups) (Table 2). In both groups, the proportions of success in a single attempt were similar (64.5% in the DPA group and 61.8% in the PTA group, *P* = .675) with equal median attempt (1) in the DPA and PTA groups, respectively (Table 2). Time of cannulation was significantly lower (median, inter quartile range [IQR]) in the DPA group as compared to the PTA group [44 (29, 75) and 37 (28, 63) seconds, respectively, *P* = .027] (Table 2; Figure 2). Ease of cannulation score was the same (median: 3) in both groups, whereas ≥4 score was 16.4% and 19.1% in the DPA and PTA groups, respectively (*P* = .596) (Table 2). There was no difference in dwell time between the 2 groups (Table 2). The reasons for cannulation failure and complications between the 2 groups were similar (Table 2). Only 5.4% and 3.6% of patients reported complications in the DPA and PTA groups, respectively (*P* = .515) (Table 2). It should be however noted that though more than 90% of patients in each group did not have any complications during the period under observation and it was statistically similar, comments regarding the difference in complications profile cannot be made due to very fewer numbers observed complications.

**Figure 3.**
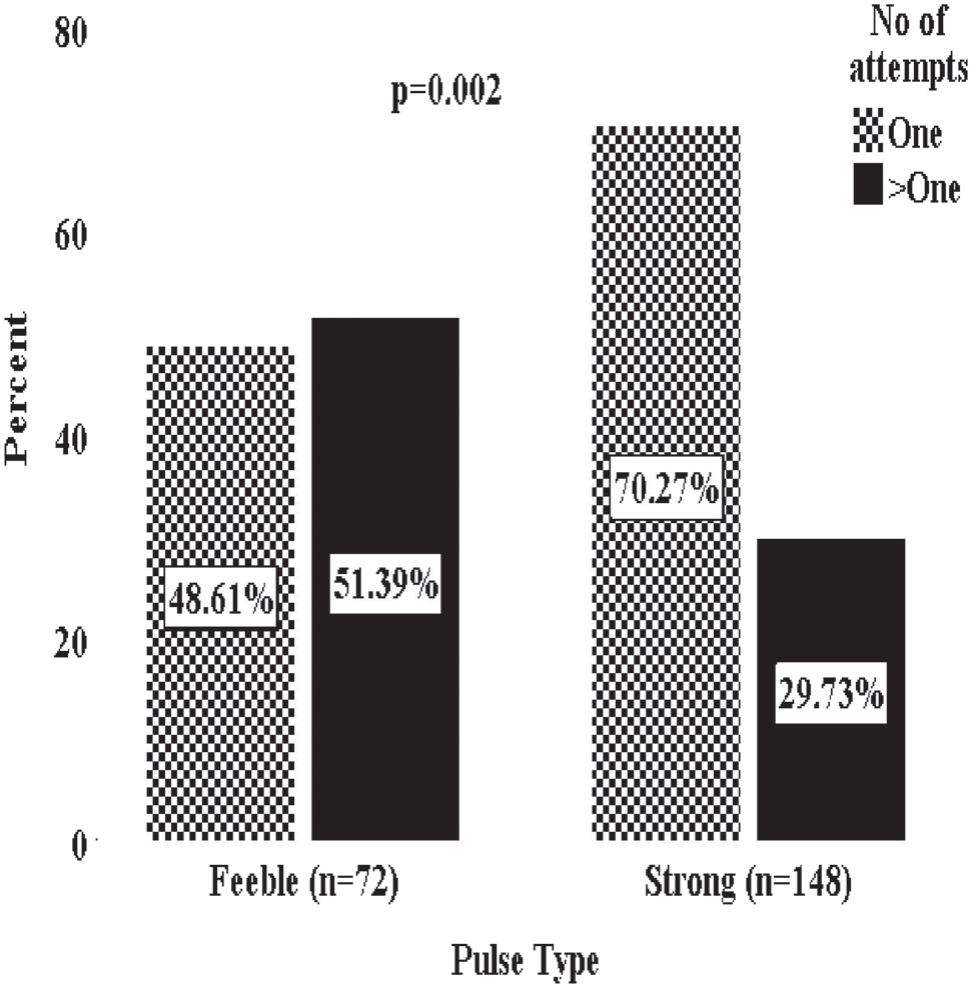
Adjacent bar diagram showing the success rates (%) of cannulation in a single attempt vs. more than 1 attempt in feeble and strong pulse groups.

**Figure 4.**
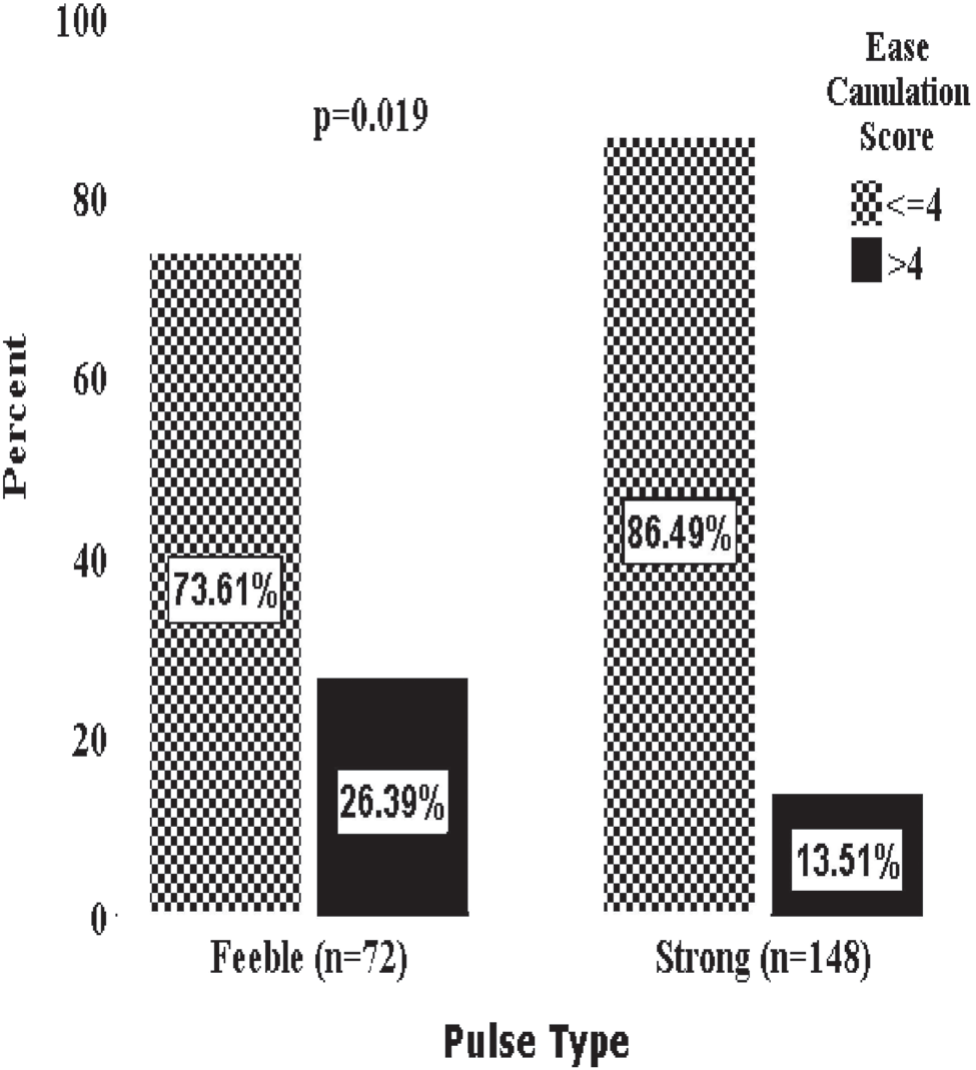
Adjacent bar diagram showing the proportion of the patient having easy cannulation score (≤4 vs. >4) in feeble and strong groups.

The association of the pulse type (feeble and strong) was assessed with the number of attempts for successful cannulation and ease of cannulation score. The success rate of cannulation in a single attempt was lower in the feeble pulse group as compared to the strong pulse group (48.61% vs. 70.27%, *P* = .002) (Figure 3). Similarly, a higher VAS of ease of cannulation (>4 score) was seen in the feeble pulse group as compared to the strong pulse group (26.39% vs. 13.51%, *P *= .019) (Figure 4).

## Discussion

Arteries of the foot are recognised sites for arterial cannulation out of which the suitability of DPA has been previously endorsed, but data pertaining to PTA cannulation in adults are scarce.^5-7^ The DPA is the extension of the anterior tibial artery, and its pulse can be palpated lateral to the extensor hallucis longus tendon (or medially to the extensor digitorum longus tendon) distal to the dorsal most prominence of the navicular bone. The DPA is considered to be the preferred site for arterial cannulation when the radial artery is inaccessible due to excellent collateral flow, easy cannulation, minimal patient inconvenience, and low incidence of complications.^12,13^ The posterior tibial artery is one of the terminal branches of the popliteal artery which is easily palpable at the midpoint between the medial malleolus and the Achilles tendon, even in the prone position.^6^ Collateral connections exist between the DPA and the lateral plantar artery (branch of PTA) to complete the plantar arch.^14^

Our observations revealed that the proportions of patients having strong pulses in the DPA and PTA were similar. The first-attempt success rate with regards to the ability to cannulate either the DPA or PTA by the conventional palpatory catheter over needle method was similar. The inability to thread the catheter inside the arterial lumen and the inability to hit the artery in equal proportions were the 2 most common reasons for the failure of the procedure in both groups. Patients with feeble pulses had a significantly lower proportion of single-attempt success rates and higher difficulty scores as compared to patients with strong pulses.

Anand et al^8^ in their study, compared the cannulation characteristics of DPA via palpatory versus ultrasound guidance (USG) and observed the first pass success, number of attempts needed, and time required for cannulation. As USG is increasingly being used for arterial cannulation, the practice of the palpatory method in our study may be questioned. It is worthwhile to note that though the use of USG for the cannulation of DPA was feasible, it was not associated with an increase in instances of higher first-attempt success rates. The use of USG also did not decrease the total number of cannulation attempts or total procedure time.^8^ The proportion of patients with successful first pass, number of attempts needed, and time for cannulation observed with USG is similar to our study.

The first-pass success rates of both the DPA as well as PTA were similar in our study. Even after an extensive literature search, we could not find any study where the characteristics of USG-guided cannulation of PTA have been evaluated in adult patients. Kim et al^11^ had however observed that in paediatric patients first-pass success rates were higher in the PTA as compared to DPA (75% vs. 45%; *P *< .001; odds ratio, 3.95; 95% CI, 1.99 to 7.87) along with shorter cannulation times [21 seconds (14, 30) vs. 34 seconds (17, 37)]. They attributed this difference to the larger size, deeper location, and ease of probe placement in the PTA group as compared to the DPA group. Although we did not use ultrasound to corroborate the findings, we, however, did not find any clinically significant difference.

Certain observations were the secondary outcomes of our study like the time required for cannulation and the complications. The time taken for cannulation of radial and ulnar artery by the palpatory method was shorter than the time required for DPA or PTA cannulation. Although our study was not designed to examine the difference in time needed to cannulate arteries of the upper versus lower limbs, deeper locations of the arteries in the lower limb may increase the time for cannulation. Authors have suggested that the short duration of cannulation may lead to decreased instances of clinically relevant thrombus formation. Though the dwell time in our study was higher compared to theirs, we also did not observe any clinically significant complications attributed to thrombosis of the vessel like colour change, digital ischaemia, or neurological defect. In terms of complications, 4.5% of patients in the DPA group and 3.6% of patients in the PTA group developed haematoma, and bleeding was observed in 0.9% of patients in the DPA group.

The time needed for cannulation of PTA when compared with DPA was significantly higher in our study. A subjective explanation as experienced by the proceduralist was that it was due to the variances in the anatomical locations. Dorsalis pedis artery being superficial and covered only by skin overlies the navicular bone which provides rigid support and prevents it from getting displaced during cannulation. In contrast, the PTA does not have a rigid bony support beneath it to prevent its lateral displacement during cannulation attempts. This explanation however requires further validation. Nonetheless, this difference of a few seconds probably has negligible clinical context. Moreover, PTA being covered by the skin, subcutaneous tissue, and flexor retinaculum is located comparatively deeper. Nakayama et al^15^ reported that the optimal depth from the skin surface is 2 to 4 mm during ultrasound-guided arterial cannulation. Whether the depth from the skin surface of the posterior tibial artery influences its cannulation characteristics needs further investigation.^11^

Our study has certain inherent limitations. First, the proceduralist was aware of the group allocation during cannulation. We, therefore, attempted to minimise the ascertainment bias by keeping the data analyst blinded to the identity of the study groups. Second, arterial cannulation was performed by an anaesthesiologist conversant with the procedure. Results obtained from this study cannot be generalised to other clinicians who do not practice these techniques routinely and would require a learning curve to achieve a similar degree of skills. Third, with ultrasound becoming the standard of care nowadays, blind techniques have been replaced. Performance variations using ultrasound can be the subject of future research. Lastly, instead of a clinical evaluation of complications, a Doppler exam of the vessels should have been performed to assess the degree of subclinical luminal effect on the vessels.

To conclude, both the DPA as well as PTA are feasible alternatives for cannulation with comparable first-attempt success rates and ease with almost minimal complication rates. However, the time taken for cannulating the PTA is significantly higher than that for DPA.

## Figures and Tables

**Table 1.** Distribution of Demographic and Clinical Variables Between Dorsalis Pedis and Posterior Tibial artery Groups (n = 220)

Variables		Dorsalis Pedis (n = 110)	Posterior Tibial (n = 110)	*P*
Age (years, mean ± SD)		39.23 ± 14.35	41.75 ± 15.62	.215
Sex (male), n (%)		51 (46.4%)	72 (65.5%)	**.004**
BMI (kg m^−2^, mean ± SD)		23.65 ± 4.13	23.62 ± 4.02	.966
ASA grade (n, %)	Grade 1	74 (67.3%)	65 (59.1%)	.208
	Grade 2	36 (32.7%)	45 (40.9%)	

BMI, body mass index; ASA, American Society of Anesthesiologists.

Independent samples *t*-test to compare means/chi-square test to compare proportions between 2 groups. *P* < .05 significant.

**Table 2.** Distribution of Clinical Variables Between Dorsalis Pedis and Posterior Tibial artery Groups (n = 220)

Variables	Dorsalis Pedis (n = 110)	Posterior Tibial (n = 110)	*P*
Pulse type (n, %) Feeble Strong	32 (29.1%)	40 (36.4%)	.250
	78 (70.9%)	70 (63.6%)	
Number of attempts Median (Q1, Q3) Single attempts	1 (1, 2)	1 (1, 2)	.709
	71 (64.5%)	68 (61.8%)	.675
Ease of cannulation Median (Q1, Q3) ≥4 score (n, %)	3 (3, 4)	3 (3, 4)	.590
	18 (16.4%)	21 (19.1%)	.596
Time of cannulation in seconds, median (Q1, Q3)	37 (28, 63)	44 (29, 75)	**.027**
Dwell time in seconds, mean ± SD	359.55 ± 96.23	359.63 ± 105.02	.995
Reason for failure of cannulation, (n, %) Inability to thread Difficulty in hitting the artery	19 (48.7%)	22 (52.4%)	.742
	20 (51.3%)	20 (47.6%)	
Complications, (n, %) Haematoma Bleeding None	5 (4.5%)	4 (3.6%)	N/A
	1 (.9%)	0 (0%)	
	104 (94.5%)	106 (96.4%)	

Independent samples *t*-test to compare means/Mann–Whitney U-test to compare medians/Chi-square test to compare proportions between 2 groups. *P* < .05 significant. N/A, not applicable: *P* value was not computed due to very small number of sample size in the bleeding group.

## References

[1] Karacalar S Ture H Baris S Karakaya D Sarihasan B . Ulnar artery versus radial artery approach for arterial cannulation: a prospective, comparative study. J Clin Anesth. 2007;19(3):209 213. (10.1016/j.jclinane.2006.10.012) 17531730

[2] Lee D Kim JY Kim HS Lee KC Lee SJ Kwak HJ . Ultrasound evaluation of the radial artery for arterial catheterization in healthy anesthetized patients. J Clin Monit Comput. 2016;30(2):215 219. (10.1007/s10877-015-9704-9) 26013978

[3] Quan Z Tian M Chi P Cao Y Li X Peng K . Modified short axis out-of-plane ultrasound versus conventional long-axis in-plane ultrasound to guide radial artery cannulation: a randomized controlled trial. Anesth Analg. 2014;119(1):163 169. (10.1213/ANE.0000000000000242) 24806143

[4] Martin C Saux P Papazian L Gouin F . Long-term arterial cannulation in ICU patients using the radial artery or dorsalis pedis artery. Chest. 2001;119(3):901 906. (10.1378/chest.119.3.901) 11243975

[5] Chen Y Cui J Sun JJ et al. Gradient between dorsalis pedis and radial arterial blood pressures during sevoflurane anaesthesia: a self-control study in patients undergoing neurosurgery. Eur J Anaesthesiol. 2016;33(2):110 117. (10.1097/EJA.0000000000000295) 26694940

[6] Rawle P Cannulation of the posterior tibial artery. Anaesthesia. 1990;45(7):589 590. (10.1111/j.1365-2044.1990.tb14840.x) 2386288

[7] Spahr RC MacDonald HM Holzman IR . Catheterization of the posterior tibial artery in the neonate. Am J Dis Child. 1979;133(9):945 946. (10.1001/archpedi.1979.02130090073014) 474547

[8] Anand RK Maitra S Ray BR et al. Comparison of ultrasound-guided versus conventional palpatory method of dorsalis pedis artery cannulation: a randomized controlled trial. Saudi J Anaesth. 2019;13(4):295 298. (10.4103/sja.SJA_766_18) 31572072 PMC6753743

[9] Johnstone RE Greenhow DE . Catheterization of the dorsalis pedis artery. Anesthesiology. 1973;39(6):654 655. (10.1097/00000542-197312000-00023) 4761027

[10] Mowlavi A Whiteman J Wilhelmi BJ Neumeister MW McLafferty R . Dorsalis pedis arterial pulse: palpation using a bony landmark. Postgrad Med J. 2002;78(926):746 747. (10.1136/pmj.78.926.746) 12509693 PMC1757948

[11] Kim EH Lee JH Song IK Kim JT Lee WJ Kim HS . Posterior tibial artery as an alternative to the radial artery for arterial cannulation site in small children: a randomized controlled study. Anesthesiology. 2017;127(3):423 431. (10.1097/ALN.0000000000001774) 28682811

[12] Gu WJ Wu XD Wang F Ma ZL Gu XP . Ultrasound guidance facilitates radial artery catheterization: a meta-analysis with trial sequential analysis of randomized controlled trials. Chest. 2016;149(1):166 179. (10.1378/chest.15-1784) 26426094

[13] Franklin CM The technique of dorsalis pedis cannulation. An overlooked option when the radial artery cannot be used. J Crit Illn. 1995;10(7):493 498. 10150581

[14] Spoerel WE Deimling P Aitken R . Direct arterial pressure monitoring from the dorsalis pedis artery. Can Anaesth Soc J. 1975;22(1):91 99. (10.1007/BF03004824) 1109711

[15] Nakayama Y Nakajima Y Sessler DI et al. A novel method for ultrasound-guided radial arterial catheterization in pediatric patients. Anesth Analg. 2014;118(5):1019 1026. (10.1213/ANE.0000000000000164) 24781571

